# Multiorgan Involvement and Particularly Liver Injury in Long COVID: A Narrative Review

**DOI:** 10.3390/life15081314

**Published:** 2025-08-19

**Authors:** Carmen-Elena Florea, Bianca Bălaș-Maftei, Alexandra Rotaru, Patricia Lorena Abudanii, Stefana Teodora Vieru, Maria Grigoriu, Adelina Stoian, Carmen Manciuc

**Affiliations:** 1 Doctoral School, “Grigore T. Popa” University of Medicine and Pharmacy, 700115 Iasi, Romania; ciornei.carmen-elena@d.umfiasi.ro (C.-E.F.); alex_rotaru2007@yahoo.com (A.R.);; 2 “Sf. Parascheva” Clinical Hospital for Infectious Diseases, 700116 Iasi, Romania; 3 Department of Medical Sciences II, “Grigore T. Popa” University of Medicine and Pharmacy, 700115 Iasi, Romania; 4 St. Mary Emergency Clinical Hospital for Children, 700309 Iasi, Romania; 5 Clinical Hospital of Pneumology, 700115 Iasi, Romania

**Keywords:** long COVID, liver injury, SARS-CoV-2 virus, COVID-19 complications

## Abstract

Since the start of the COVID-19 pandemic, increasing evidence has shown that SARS-CoV-2 infection can cause long-term symptoms, collectively known as long COVID, and that patients with mild COVID-19 can also be affected by persistent fatigue, cognitive impairment, dyspnea, muscle pain, etc. Recent research has also found multiple organ systems, including the liver, to be significant sites of ongoing injury. This narrative review summarizes current knowledge on organ involvement during and after COVID-19, with particular focus on early and delayed hepatic manifestations and associated risk factors. Pathogenesis appears to be multifactorial, involving direct virus action, the body’s immune-mediated inflammatory response, microvascular damage, drug-induced hepatotoxicity, and, in some cases, reactivation or exacerbation of pre-existing liver conditions. The hepatic clinical manifestations range from asymptomatic elevations of transaminases to cholangiopathy and even fibrosis. These can persist or progress for months after the initial infection with SARS-CoV-2 is resolved, requiring prolonged monitoring and interdisciplinary care, especially in the presence of metabolic disorders, obesity, or hepatitis. Neurological, cardiovascular, and other sequelae are discussed in parallel, with attention paid to common inflammatory and thrombotic pathways. This review concludes that liver dysfunction is of particular interest in long-COVID due to the liver’s central role in metabolism and inflammation. While further research is being conducted into organ-specific and systemic interactions, the available evidence makes a compelling case for extended monitoring and integrated management strategies post infection.

## 1. Introduction

SARS-CoV-2, the virus that causes COVID-19, enters the body through angiotensin-converting enzyme-2 (ACE2) receptors, which are common in many types of cell making up the lungs, livers, gastrointestinal tract, brain, and heart. Even when the initial infection is mild or moderate, some individuals experience effects that can last for months or even longer. The term “long COVID” was therefore introduced to describe symptoms that persist and reoccur after a patient recovers from the acute phase of a SARS-CoV-2 infection. These can include extreme fatigue, difficulty breathing, muscle pain, cognitive issues often referred to as “brain fog”, etc., as long COVID can affect different body systems, including the cardiovascular, neurological, respiratory, and gastrointestinal systems [1].

According to the World Health Organization (WHO), long COVID typically manifests approximately three months after an acute COVID-19 illness, whenever it cannot be explained by other diagnoses. With millions of people affected worldwide, mostly female and over the age of 70, long COVID has made its way onto the public health agenda as a serious issue. In the UK, for instance, the Office for National Statistics estimated that the prevalence of symptoms lasting beyond 12 weeks ranges from 3% to 11.7%, with a significant impact on individuals’ daily routines, as well as on their social and professional lives [2].

The mechanisms behind long COVID and potential treatments for it are new areas of research prompted by increasing awareness and concern [3]. This review aims to synthesize the current evidence of multiorgan impact in long COVID, with a particular focus on the mechanisms, manifestations, and risk factors associated with liver injury.

## 2. Materials and Methods

The PubMed database was searched for any articles published between March 2020 and December 2023 mentioning “long COVID”, “liver injury”, or “SARS-COV-2 and liver injury”. No other search filters were applied at this stage. The results were screened to select and review studies focusing more specifically on long COVID. More than 500 articles were identified initially, of which 25 were selected for the purposes of this review of early research on long COVID. These were chosen based on the level of detail and comprehensiveness in their analysis. Newer references were added later, during the manuscript peer-review process, to facilitate readership.

## 3. SARS-CoV-2 Virus Structure and Mechanism of Infection

The SARS-CoV2, SARS-CoV, and MERS-CoV viruses belong to the *Coronaviridae* family. All are enveloped, single-stranded RNA viruses. The SARS-CoV-2 virus is a spherical virus with a diameter of approximately 60–140 nanometers. Its surface is studded with spike-like projections, giving it the appearance of a solar corona under the electron microscope, hence the name “coronavirus”. The viral envelope is composed of a lipid bilayer derived from the host cell membrane, into which structural proteins are embedded [4,5]. The virus genome is composed of a single strand of positive-sense RNA approximately 29.9 kilobases in length, making it one of the largest genomes among RNA viruses. This genome encodes for both structural and non-structural proteins. Four major structural proteins are essential to the architecture and function of the virus:

1. Spike protein—responsible for binding to the host cell receptor (ACE2) and facilitating viral entry. This protein is also the primary target for neutralizing antibodies and vaccine development.
2. Envelope protein—involved in virus assembly, budding, and release.
3. Membrane protein—the most abundant structural protein, playing a central role in shaping the viral envelope and coordinating virus assembly.
4. Nucleocapsid protein—encapsulates the viral RNA genome, aiding in its stability and packaging into new virions [6,7].

The infection process begins when the Spike protein binds to the angiotensin-converting enzyme 2 (ACE2) receptors on the surface of cells. Systemic infection occurs due to widespread ACE2 expression across organ systems, including the lungs, liver and digestive tract, heart, muscles, nervous system, pancreas, etc. This interaction is facilitated by a host cell enzyme, TMPRSS2, which primes the Spike protein, allowing the viral and cellular membranes to fuse. Once inside the cell, the viral RNA is released and hijacks the host’s ribosomes to produce viral proteins. Simultaneously, the virus replicates its RNA genome through a complex involving viral enzymes such as RNA-dependent RNA polymerase. The newly synthesized viral proteins and RNA genomes are assembled into mature virions within the endoplasmic reticulum–Golgi intermediate compartment (ERGIC). These virions are then transported to the cell membrane and released through exocytosis to infect neighboring cells [8,9].

## 4. Immune-Mediated Severity in COVID-19, Inflammatory Response, and Subsequent Organ Damage

In the context of SARS-CoV-2 infection, the immune system can enter a state of hyperactivation, particularly involving cytotoxic T cells and other immune cells responsible for the humoral immune response. Exaggerated immune responses have been found to play a critical role in the development of multiple organ failure (MOF) in patients with severe COVID-19. This excessive reaction is commonly referred to as a *cytokine storm*, characterized by the massive release of pro-inflammatory cytokines, including interleukin-6 (IL-6), TNF-alpha, and others. These are proteins that normally regulate the immune response; in excessive amounts, they cause significant inflammation and tissue damage [10,11,12].

Autopsy studies conducted on patients who succumbed to COVID-19 have revealed that this hyperactivation of cytotoxic T cells and the excessive release of cytokines contribute to tissue damage and the onset of dysfunction across multiple organs, eventually leading to MOF. Interleukin-6 (IL-6), in particular, has been identified as a useful biomarker for predicting severe forms of the disease, with elevated levels being associated with an increased risk of developing multiple organ dysfunction [13]. In summary, in severe and critical cases of COVID-19, the uncontrolled immune response, including hyperactivation of both humoral and cellular pathways, plays a key role in the body’s deterioration and the development of multiple organ failure, complicating the management and treatment of these patients.

### Ferritin as an Inflammatory Marker and Prognostic Indicator

Harmful levels of inflammation can also be expressed as elevated ferritin in the blood. It is believed that cytokines stimulate ferritin production as part of the body’s response to oxidative stress and inflammation. Ferritin itself also has immunomodulatory properties, playing a role in regulating inflammation, but excessively high levels can contribute to tissue damage and organ dysfunction [14,15].

Increased ferritin levels are not unique to COVID-19; they occur in many other inflammatory or infectious conditions. However, in COVID-19, patients with severe or critical forms of the disease tend to have much higher ferritin levels compared to mild or moderate cases. This may explain the clinical presentation in patients who rapidly progress to respiratory failure and other major complications.

Studies have shown that excessively high ferritin levels (over 500 μg/L or higher) are associated with an increased risk of respiratory failure, acute respiratory distress syndrome (ARDS), sepsis, and even death. Additionally, an elevated ferritin level has also been linked to coagulopathy (blood clotting disorders), commonly observed in COVID-19 patients. This makes ferritin a useful marker for understanding and assessing the severity of the disease and for monitoring hospitalized patients [16,17].

## 5. Long COVID Symptoms and Affected Organ Systems

**Figure 1:**
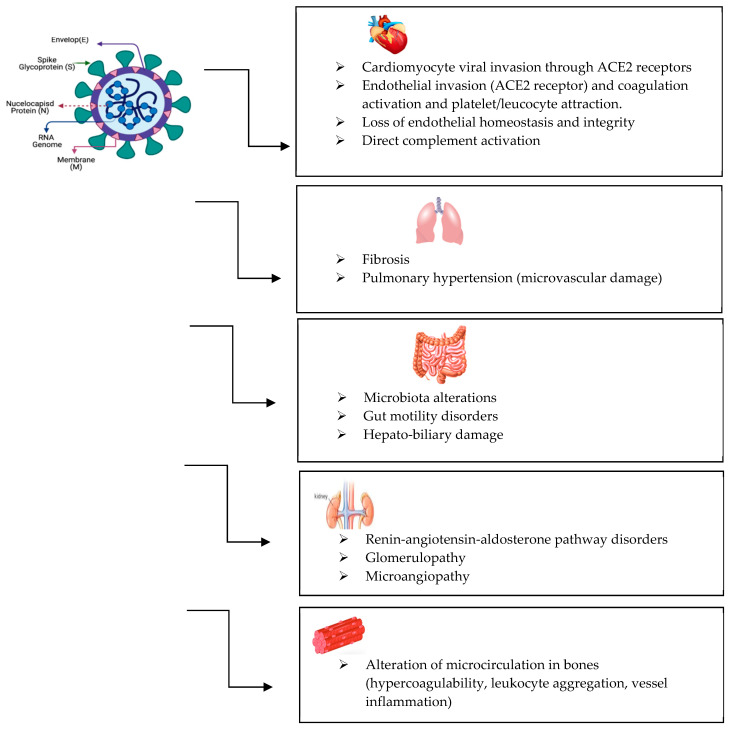
Multiorgan involvement in long COVID.

It is difficult to know how many patients have had long COVID because many did or do not seek follow-up examination after the initial infection, with or without new or persisting symptoms [2]. Initially, long COVID was attributed to severe forms of acute SARS-CoV-2 infection or with hypoxia, but this theory was dropped when patients who had never required hospitalization for COVID-19 started reporting a wide range of long-term symptoms, prompting research that is still ongoing. In fact, patients may develop long COVID regardless of the severity of their acute infection episode. One hypothesis is that some of the viral RNA may remain active in the body cells, yet this alone cannot account for how the different organ systems are affected, as summarized below (see also Figure 1). Available studies suggest that male gender and advanced age increase the risk of long COVID, along with pre-existing cardiovascular and respiratory conditions [18,19].

Fatigue is the most common general symptom, and patients describe it as more than tiredness, as lack of energy is accompanied or amplified by loss of motivation and concentration. This kind of fatigue specific to long COVID has been compared to chronic fatigue syndrome and myalgic encephalomyelitis, which share psychiatric and neurological symptoms. Several mechanisms have been thought to cause fatigue, such as hypometabolism in the frontal lobe and cerebellum and the congestion of the glymphatic system influencing resistance to cerebrospinal fluid drainage through the cribriform [20]. Another theory is that muscular fibers and neuromuscular junctions may become inflamed under direct action from the SARS-CoV-2 virus. Negative social and psychological factors have also been suggested to play a role.

The most severe short- and long-term effects of COVID-19 caused by infection with the SARS-CoV-2 virus are seen in the lungs, and so the most common specific symptom in long COVID is dyspnea. Older patients, patients who required a long period of hospitalization, and those with pulmonary comorbidities are more likely to develop fibrotic transformations of the lungs. The destruction of endothelial cells by the novel coronavirus is one possible mechanism, and fibrotic processes affecting lung tissue may be facilitated further by cytokines such as interleukin-6 [21]. Pulmonary dysfunction has also been associated with high levels of D-dimer and blood urea nitrogen [22]. However, dyspnea occurring well after the acute infection does not seem intrinsically linked to acute respiratory failure or the severity of the acute phase. In some cases, persistent dyspnea was reported more than 2 months after the initial illness [1].

With heart cells featuring high numbers of ACE2 receptors, the virus enters and causes fragmentation and enucleation, so cardiac injury is common in SARS-CoV-2 infection [23]. Elevated levels of interleukin-6, C-reactive protein (CRP), neutrophils, and procalcitonin have been linked to heart and kidney dysfunction [24,25]. Increased troponin levels also indicate initial myocardial inflammation, which can later develop into myocarditis and cardiovascular abnormalities that are not explained by age and other known factors. Residual myocarditis has occurred in patients who are young or practice a sport. Additionally, a new pathological entity was observed and called postural orthostatic tachycardia syndrome. Another mechanism which can produce cardiovascular abnormalities in long COVID is related to microthrombosis and endothelial damage [26,27].

Patients have also complained of headache, cognitive impairment, seizures, altered mental status, and especially “brain fog”. Such manifestations can be a consequence of the cytokine storm and the activation of glial cells. The permeability of the blood–brain barrier is affected in acute infection, allowing cytokines to enter the brain and produce neuroinflammation. Evidence of neurological involvement includes inflammation in the cerebrospinal fluid, and MRI examinations have shown the frontal lobe to be the part of the brain most affected in long COVID. At the same time, symptoms such as extreme fatigue and cognitive impairment can be explained as effects of microvascular damage. Cardiovascular abnormalities such as hypercoagulability and cardio embolism can even cause a stroke.

The SARS-CoV-2 virus is also capable of entering the neurological system via olfactory neurons and the mucous membrane of the nose and oral cavity, affecting the sense of smell and taste. The deterioration of gustatory particles is a negative outcome of the interaction between the SARS-CoV-2 virus and sialic acid receptors, potentially leading to ageusia [28].

Moreover, social isolation during quarantine increased the risk to develop depression, anxiety, and post-traumatic stress disorder, especially for institutionalized patients with established dementia. Sleep disorders were amplified by loneliness, and sleeping pills were the most prescribed, after antipyretics and antitussives [1]. It was also observed that patients with psychiatric comorbidities are more likely to develop long COVID [29].

Other forms of long COVID can involve acute kidney injury, leading to decreased kidney function even six months after infection, or high initial levels of amylase and lipase, leading to acute pancreatitis. The pancreas has even more ACE2 receptors than the lungs, which may explain why it is often affected, but the mechanism is not yet well known (whether it is a direct effect of the virus or an indirect response to inflammation). Similarly, the spleen also contains ACE2 receptors and patients have presented with splenic infarction and severe lymphocytopenia explained by the atrophy of lymphoid follicles. Last but not least, in cases where antibiotics were heavily used to address infection, this caused changes in the gut microbiota and facilitated secondary infection with *Clostridioides difficile*, in addition to direct viral action through the ACE2 receptors in the gastrointestinal tract [30,31,32,33].

## 6. Liver Involvement in COVID-19 and Long COVID

Hepatic involvement in COVID-19 is defined as any type of liver injury that occurs at any of the stages of SARS-CoV-2 infection, including during the treatment of infection, and excluding prior evidence of liver disease.

The main pathological mechanisms cited in disease formation and progression include the direct cytotoxic effects resulting from active intracellular replication in the liver, hypoxia secondary to acute respiratory failure, immune-mediated liver damage resulting from severe systemic inflammatory response, vascular dysfunction resulting from coagulopathy, endothelin exposure, or via right heart failure, treatment-related liver injury, and exacerbation of pre-existing liver disease [34]. Table 1 summarizes some of the initial and delayed liver complications, illustrating the complex effects of the SARS-CoV-2 virus on the human body.

The initial inflammatory response and the more severe cytokine storms that occur in COVID-19 can lead to liver damage and hepatic symptoms both during the acute illness and long after the acute SARS-CoV-2 infection has been resolved. The use of medications during the acute phase, such as antivirals, corticosteroids, or immunosuppressants, may place further stress on the liver, contributing to elevated transaminases as well. In some individuals, increased ALT and AST levels can be due to overactive immune responses and persistent systemic inflammation [35,36].

Some patients with long COVID continue to experience liver dysfunction, as evidenced by elevated transaminase and lactate dehydrogenase (LDH) levels. This has been observed in approximately 15–65% of COVID patients. Additionally, hypoalbuminemia, while a nonspecific marker, has been associated with negative prognosis [20,21,23]. Post-COVID-19, cholangiopathy is a noteworthy complication that involves elevated liver markers (alkaline phosphatase, bilirubin, transaminases) and bile duct abnormalities on imaging. A review of reported cases up to the end of 2021 suggests that patients who had COVID-19, especially those hospitalized, should be monitored by a multidisciplinary team for at least six months [37,38].

In an analysis of 150 autopsies at Semmelweis University in Hungary, liver tissue samples from 20 deceased patients displayed steatosis, necrosis, fibrosis, and inflammation, with sinusoidal ectasia and endothelial damage common in all cases. SARS-CoV-2 RNA and proteins were found in liver non-parenchymal cells, such as endothelial and Kupffer cells, but not in hepatocytes. Endothelial damage was the most frequent liver alteration, indicating a role in liver disease associated with COVID-19. Patients recovering from severe COVID-19 may therefore face prolonged liver repair and should be monitored regularly post-infection [39,40].

Other research showed that liver enzyme levels and the FIB-4 index rise during acute COVID-19, even without pre-existing liver conditions, suggesting increased liver fibrosis. One study assessed liver fibrosis using serum hyaluronic acid (HA) and FIB-4 in acute COVID-19 patients, post-COVID patients, and a control group. In the acute group, 65% of patients had elevated FIB-4 scores and 54% had increased HA levels. Both HA and FIB-4 correlated with liver function and inflammatory markers. In the post-COVID group, 5% had signs of liver fibrosis. These findings highlight the potential for liver fibrosis not only during but also after COVID-19 [39].

### Pre-Existing Conditions and Other Risk Factors for Liver Injury During/After COVID-19

A meta-analysis of early data found that metabolic dysfunction-associated fatty liver disease (MAFLD) might increase the risk of severe COVID-19, requiring hospitalization and ICU admission, but not mortality. Individuals with both MAFLD and obesity were six times more likely to develop severe COVID-19, and liver fibrosis predicted adverse outcomes. Raised AST levels predicted mortality from COVID-19, though liver injury mechanisms remain unclear. Autopsies showed steatosis, vascular thrombosis, and liver fibrosis in deceased COVID-19 patients. Higher FIB-4 scores were linked to lower IgG levels after vaccination, even though using the FIB-4 score to assess fibrosis was methodologically limited due to the illness altering liver enzymes. Monitoring glucose and liver function in MAFLD patients with COVID-19 was deemed crucial [41].

In turn, SARS-CoV-2 infection was found to possibly aggravate pre-existing liver conditions, leading to chronic transaminase elevation even after recovery from the initial illness [42,43]. In patients with histories of hepatitis, COVID-19 can increase both the severity of liver disease and viral replication. The interaction between the SARS-CoV-2 virus and pre-existing viral hepatitis B or C may exacerbate liver damage, leading to worse outcomes and heightened risk of complications. The immune dysregulation caused by COVID-19 may facilitate increased viral replication of the hepatitis virus, further aggravating the patient’s condition. Moreover, the immune response triggered by COVID-19 can lead to the development of autoimmune hepatitis, where the immune system attacks healthy liver cells [44].

Other liver conditions which COVID-19 can worsen include metabolic stress and non-alcoholic fatty liver disease (NAFLD), particularly in individuals with clinical obesity and/or metabolic syndrome. The viral infection can exacerbate fat accumulation in the liver, potentially leading to inflammation (steatohepatitis) [45]. Liver scarring, or fibrosis, is yet another possible negative outcome of persistent inflammation, increasing the risk of cirrhosis as a long-term complication.

Furthermore, hepatic injury and complications after COVID-19 can be triggered by previously unidentified genetic predisposition for hepatic pathologies, as well as by behavioral factors such as alcohol consumption. Additionally, patients with diabetes mellitus and obesity may develop liver injuries in the context of long COVID [46].

## 7. Monitoring, Diagnosis, and Treatment of Long COVID

Managing long COVID is complex and often requires a multidisciplinary approach, with liver health being a key consideration. Individuals with long COVID who show signs of liver dysfunction need regular blood tests to assess liver function and imaging studies if necessary. Treatment may include lifestyle changes (diet, exercise) and medications to control inflammation or underlying conditions. Careful monitoring and timely medical intervention are essential to prevent serious complications [13].

Long COVID is a challenging pathology, and the international medical community is still working towards the most effective treatments. With such heterogenous manifestations as we have reviewed above, it is clear that patients require a personalized approach. Current guidelines recommend follow-up assessment of COVID-19 patients four weeks after the acute episode, and tests including hemoleukograms, inflammation markers, renal and hepatic function tests, vitamin levels (especially D and B group), and ferritin levels, along with other tests to identify mental and physical dysfunction [47].

To prevent or counteract chronic inflammation, persistent fatigue, and cognitive dysfunction typical in long COVID, supplements containing multivitamins are recommended, such as from the B group, and products with Coenzyme Q10. In cases of mast cell activation syndrome, antihistamines may be used with caution to avoid dopamine fluctuations that have been associated with the onset of dementia later on [3].

## 8. Conclusions

Long COVID is still a new area of research, but there is now sufficient evidence to highlight its clinical and pathophysiological complexity, leading to its wide-ranging and debilitating symptoms even in mild cases of initial COVID-19 illness. Within the broader multisystem dysfunction, the liver is particularly vulnerable to the convergence of viral, immunological, vascular, and metabolic mechanisms. Liver involvement in long COVID is not uncommon and can have long-term consequences, especially in patients with pre-existing risk factors. Liver function should be routinely monitored in the months following acute SARS-CoV-2 infection to prevent and mitigate prolonged or progressive liver damage.

## Figures and Tables

**Table 1:** Mechanisms that may contribute to liver damage in SARS-CoV-2 infection.

Direct cytopathy	Virus invading liver cells => cytopathic effects => liver dysfunction
Immune-mediated damage	Virus infection => dysregulated inflammatory response characterized by increased pro-inflammatory cytokines => severe liver dysfunction
Hypoxia/ischemia (in severe cases)	Multiple organ dysfunction => hypoxia-related acute respiratory distress syndrome, hypotension, or congestive heart failure => liver dysfunction
Microvascular thrombosis	Impaired blood flow to the liver => further exacerbation of liver injury
